# Strategies to increase the demand for childhood vaccination in low- and middle-income countries: a systematic review and meta-analysis

**DOI:** 10.2471/BLT.14.146951

**Published:** 2015-03-23

**Authors:** Mira Johri, Myriam Cielo Pérez, Catherine Arsenault, Jitendar K Sharma, Nitika Pant Pai, Smriti Pahwa, Marie-Pierre Sylvestre

**Affiliations:** a Centre de Recherche du Centre Hospitalier de l’Université de Montréal (CRCHUM), Tour Saint-Antoine, Porte S03-458, 850 rue St-Denis, Montréal, Québec, H2X 0A9, Canada.; b Département d’administration de la santé, Université de Montréal, Montréal, Canada.; c Department of Epidemiology, Biostatistics and Occupational Health, McGill University, Montréal, Canada.; d National Health Systems Resource Centre (NHSRC), Ministry of Health and Family Welfare, New Delhi, India.; e Department of Medicine, McGill University, Montréal, Canada.; f Pratham Education Foundation (ASER Centre), New Delhi, India.

## Abstract

**Objective:**

To investigate which strategies to increase demand for vaccination are effective in increasing child vaccine coverage in low- and middle-income countries.

**Methods:**

We searched MEDLINE, EMBASE, Cochrane library, POPLINE, ECONLIT, CINAHL, LILACS, BDSP, Web of Science and Scopus databases for relevant studies, published in English, French, German, Hindi, Portuguese and Spanish up to 25 March 2014. We included studies of interventions intended to increase demand for routine childhood vaccination. Studies were eligible if conducted in low- and middle-income countries and employing a randomized controlled trial, non-randomized controlled trial, controlled before-and-after or interrupted time series design. We estimated risk of bias using Cochrane collaboration guidelines and performed random-effects meta-analysis.

**Findings:**

We identified 11 studies comprising four randomized controlled trials, six cluster randomized controlled trials and one controlled before-and-after study published in English between 1996 and 2013. Participants were generally parents of young children exposed to an eligible intervention. Six studies demonstrated low risk of bias and five studies had moderate to high risk of bias. We conducted a pooled analysis considering all 11 studies, with data from 11 512 participants. Demand-side interventions were associated with significantly higher receipt of vaccines, relative risk (RR): 1.30, (95% confidence interval, CI: 1.17–1.44). Subgroup analyses also demonstrated significant effects of seven education and knowledge translation studies, RR: 1.40 (95% CI: 1.20–1.63) and of four studies which used incentives, RR: 1.28 (95% CI: 1.12–1.45).

**Conclusion:**

Demand-side interventions lead to significant gains in child vaccination coverage in low- and middle-income countries. Educational approaches and use of incentives were both effective strategies.

## Résumé

**Objectif:**

Déterminer, parmi les stratégies visant à accroître la demande de vaccination, celles qui sont efficaces pour augmenter la couverture vaccinale des enfants dans les pays à revenu faible et intermédiaire.

**Méthodes:**

Nous avons recherché, dans les bases de données MEDLINE, EMBASE, de la bibliothèque Cochrane, POPLINE, ECONLIT, CINAHL, LILACS, BDSP, Web of Science et Scopus, des études à ce sujet, publiées en allemand, en anglais, en espagnol, en français, en hindi, et en portugais, jusqu'au 25 mars 2014. Nous avons inclus les études relatives à des interventions visant à accroître la demande de vaccination systématique des enfants. Les études étaient recevables si elles avaient été menées dans des pays à revenu faible et intermédiaire et si elles étaient de type essai contrôlé randomisé, essai contrôlé non randomisé, contrôle avant et après ou étude de séries temporelles interrompues. Nous avons estimé le risque de biais suivant les directives de la collaboration Cochrane et avons réalisé une méta-analyse à effets aléatoires.

**Résultats:**

Nous avons relevé 11 études, comprenant quatre essais contrôlés randomisés, six essais contrôlés randomisés en grappes et une étude contrôlée avant et après, qui ont été publiées en anglais entre 1996 et 2013. Les participants étaient généralement les parents de jeunes enfants exposés à une intervention éligible. Six études montraient un faible risque de biais et cinq études avaient un risque de biais moyen à élevé. Nous avons réalisé une analyse combinée en tenant compte des 11 études, avec des données provenant de 11 512 participants. Les interventions axées sur la demande étaient associées à une administration de vaccins sensiblement plus élevée, risque relatif (RR) : 1,30; intervalle de confiance de 95%, IC : 1,17-1,44). L'analyse de sous-groupes a également montré des effets importants pour sept études décrivant des interventions d'éducation ou d'application des connaissances, RR : 1,40 (IC de 95% : 1,20–1,63) et pour quatre études décrivant le recours à des incitations, RR : 1,28 (IC de 95% : 1,12-1,45).

**Conclusion:**

Les interventions axées sur la demande entraînent des avantages notables pour la vaccination des enfants dans les pays à revenu faible et intermédiaire. Les démarches éducatives ainsi que le recours à des incitations étaient tous deux des stratégies efficaces.

## Resumen

**Objetivo:**

Investigar qué estrategias para incrementar la demanda de vacunación son efectivas en el incremento de la cobertura de vacunación infantil en países de ingresos bajos y medios.

**Métodos:**

Se realizaron búsquedas en las bases de datos de MEDLINE, EMBASE, Cochrane Library, POPLINE, ECONLIT, CINAHL, LILACS, BDSP, Web of Science y Scopus para encontrar estudios pertinentes, publicados en alemán, español, francés, hindi, inglés y portugués hasta el 25 de marzo de 2014. Se incluyeron estudios de intervenciones con el objetivo de incrementar la demanda de vacunación infantil sistemática. Los estudios fueron elegibles si se realizaron en países de ingresos bajos y medios y utilizaron un diseño de ensayo controlado aleatorizado, ensayo controlado no aleatorizado, controlado antes y después o de series temporales interrumpidas. Se estimó un riesgo de sesgo mediante las directrices de colaboración de Cochrane y se realizaron metanálisis de efectos aleatorios.

**Resultados:**

Se identificaron 11 estudios que abarcan cuatro ensayos controlados aleatorizados, seis ensayos controlados aleatorizados por conglomerados y un estudio controlado antes y después, publicados en inglés entre 1996 y 2013. En general, los participantes fueron padres de niños pequeños expuestos a una intervención elegible. Seis estudios demostraron un bajo riesgo de sesgo y cinco estudios presentaron un riesgo de sesgo entre moderado y elevado. Se realizó un análisis agrupado teniendo en cuenta los 11 estudios, con datos de 11,512 participantes. Las intervenciones que afectan a la demanda se relacionaron con una recepción de las vacunas significativamente superior, riesgo relativo (RR): 1,30, (intervalo de confianza, IC, del 95%: 1,17–1,44). Los análisis de los subgrupos también demostraron efectos importantes de siete estudios de educación y traslación de conocimientos, RR: 1,40 (IC del 95%: 1,20–1,63) y de cuatro estudios que utilizaron incentivos, RR: 1,28 (IC del 95%: 1,12–1,45).

**Conclusión:**

Las intervenciones que afectan a la demanda llevan a mejoras significativas en la cobertura de vacunación infantil en países de ingresos bajos y medios. Los enfoques educativos y el uso de incentivos fueron estrategias efectivas.

## ملخص

**الغرض:**

استقصاء لتحديد الاستراتيجيات الفعالة في زيادة الطلب على التغطية بخدمات تمنيع الأطفال في البلدان المنخفضة الدخل والمتوسطة الدخل.

**الطريقة:**

بحثنا في قواعد معطيات MEDLINE وEMBASE ومكتبة Cochrane وPOPLINE وECONLIT وCINAHL وLILACS وBDSP وويب العلوم وScopus عن دراسات ذات صلة، منشورة باللغة الإنجليزية والفرنسية والأسبانية والبرتغالية والألمانية والهندية، حتى 25 مارس 2014. ضممنا دراسات التدخل التي تهدف إلى زيادة الطلب على تمنيع الأطفال الروتيني. تصبح الدراسات مؤهلة في حالة إجرائها في البلدان المنخفضة الدخل والمتوسطة الدخل، وإذا اعتمدت على تجربة معشاة مضبطة بالشواهد، أو تجربة غير معشاة مضبطة بالشواهد، أو تجربة مضبطة لما قبل ولما بعد، أو تصميم سلاسل زمنية متقطِّعة. قمنا بتقدير اختطار التحيز باستخدام الدلائل الإرشادية التعاونية لمكتبة Cochrane، وأجرينا تحليلاً تلوياً للآثار العشوائية.

**النتائج:**

توصلنا إلى 11 دراسة تتألف من أربع تجارب معشاة مضبطة بالشواهد، وست تجارب عنقودية معشاة مضبطة بالشواهد، ودراسة واحدة مضبطة لما قبل ولما بعد، منشورة باللغة الإنجليزية ما بين 1996 و2013. كان المشاركون في الأغلب آباءً لصغار الأطفال المعرضين لتدخل مؤهل. أظهرت ست دراسات مستوى منخفض من اختطار التحيز، وكان لدى خمس دراسات مستوى متوسط إلى مرتفع من اختطار التحيز. أجرينا تحليلاً تجميعياً مع الأخذ في الاعتبار جميع الدراسات الإحدى عشر، مع بيانات من 11512 مشاركاً. ارتبطت التدخلات من جانب الطلب بارتفاع تلقي اللقاحات بشكل ملحوظ، الاختطار النسبي: 1.30؛ (بنسبة أرجحية مقدارها 95%: 1.17 إلى 1.44).كما أظهرت تحليلات المجموعات الفرعية آثاراً ملحوظة لسبع دراسات ترجمة تعليمية ومعرفية، الاختطار النسبي: 1.40 (بنسبة أرجحية مقدارها 95%: 1.20 إلى 1.63) ولأربع دراسات استخدمت الحوافز، الاختطار النسبي: 1.28 (بنسبة أرجحية مقدارها 95%: 1.12 إلى 1.45).

**الاستنتاج:**

أدت التدخلات من جانب الطلب إلى مكاسب ملحوظة في مجال التغطية بخدمات تمنيع الأطفال في البلدان المنخفضة الدخل والمتوسطة الدخل. كانت كل من الأساليب التعليمية واستخدام الحوافز استراتيجيات فعالة.

## 摘要

**目的:**

旨在调查何种增加疫苗接种需求的策略能够有效提高中低收入水平国家中儿童接种疫苗的覆盖率。

**方法:**

我们搜索了美国联机医学文献分析和检索系统 (MEDLINE)、荷兰医学文摘数据库 (EMBASE)、考克兰 (Cochrane) 图书库、人口信息数据库 (POPLINE)、经济学文献资料库 (ECONLIT)、护理学数据库 (CINAHL)、拉丁美洲和加勒比健康科学文献库 (LILACS)、公共卫生数据库 (BDSP)、科学网 (Web of Science) 以及斯高帕斯 (Scopus) 数据库,旨在查找以英语、法语、西班牙语、葡萄牙语、德语和印地语出版的相关研究资料,这项工作的时间将一直持续到 2014 年 3 月 25 日。我们囊括了意在提高常规儿童接种疫苗需求的干预研究。如果研究是在中低收入水平的国家开展,并且开展时采用的方法为随机对照试验、非随机对照试验、前后对照或间断时间序列设计,则该研究符合条件。我们通过科克伦协作网 (Cochrane collaboration) 指南评估了偏倚风险,并且进行了随机荟萃分析。

**结果:**

我们在 1996 年和 2013 年期间鉴定了 11 项英文版研究,其中包括四项随机对照试验、六项整群随机对照试验和一项前后对照研究。参与者一般为接受合格干预的幼儿的父母。六项研究的偏倚风险较低,而五项研究具有中高级偏倚风险。我们针对从 11 512 名参与者处获得数据的 11 项研究全面开展汇整分析。需求方面的干预措施会大大提高疫苗接种率,相对风险 (RR) 为:1.30,(95% 置信区间,CI:1.17–1.44)。小群组分析也论证了七项教育和知识翻译研究的重要作用,RR 为:1.40 (95% CI:1.20–1.63),以及四项采用激励措施的研究的重要作用,RR 为:1.28 (95% CI: 1.12–1.45)。

**结论:**

需求方面的干预措施能够大大提高中低收入水平的国家中儿童接种疫苗覆盖率的成果。教育方法和使用激励措施都是有效的策略。

## Резюме

**Цель:**

Определить, какие стратегии для увеличения спроса на вакцинацию оказываются эффективными и позволяют расширить охват вакцинацией детей в странах с низким и средним уровнем доходов.

**Методы:**

Был выполнен поиск исследований, опубликованных на английском, французском, испанском, португальском, немецком языках и хинди до 25 марта 2014 г., в базах данных MEDLINE, EMBASE, Кокрановской библиотеки, POPLINE, ECONLIT, CINAHL, LILACS, BDSP, Web of Science и Scopus. Поиск охватывал исследования интервенций, направленных на повышение спроса на плановую вакцинацию детей. Отбирались исследования, которые проводились в странах с низким и средним уровнем доходов и включали рандомизированные контролируемые испытания, нерандомизированные контролируемые испытания, испытания, контролируемые перед началом и после окончания, и испытания методом прерванного временного ряда. Оценка риска системной ошибки осуществлялась согласно руководящим принципам организации «Кокрановское сотрудничество». Кроме того, был проведен метаанализ по модели со случайными эффектами.

**Результаты:**

Были выявлены 11 исследований, опубликованных на английском языке в 1996–2013 гг.: четыре рандомизированных контролируемых исследования, шесть кластерных рандомизированных контролируемых исследований и одно исследование, контролируемое перед началом и после окончания. Участники этих исследований — это в основном родители маленьких детей, в отношении которых применялась оправданная интервенция. Шесть исследований показали низкий риск системной ошибки. Другие пять — умеренный и высокий риск системной ошибки. Был проведен объединенный анализ с учетом всех 11 исследований, включающий данные 11 512 участников. Интервенции, направленные на повышение спроса, были связаны со значительно более активным согласием на вакцинацию. Относительный риск (ОР) составил 1,30 (95 % доверительный интервал, ДИ: 1,17–1,44). Анализ в подгруппах также продемонстрировал значительный эффект в семи исследованиях, связанных с информированием и передачей знаний, ОР: 1,40 (95 % ДИ: 1,20–1,63), и в четырех исследованиях, в которых использовались льготы, ОР: 1,28 (95 % ДИ: 1,12–1,45).

**Вывод:**

Интервенции, направленные на повышение спроса на вакцинацию, приводят к значительному расширению охвата вакцинацией детей в странах с низким и средним уровнем доходов. Свою эффективность показали как стратегии информирования, так и использование льгот.

## Introduction

Almost 40 years after the launch of the World Health Organization’s (WHO’s) Expanded Programme on Immunization, one in five children worldwide still does not have access to basic vaccines.^1^^,^^2^ In May 2012, the World Health Assembly approved the Global Vaccine Action Plan to ensure that the full benefits of immunization are extended to people in every region, country and community.^1^ Midway through the Global Vaccine Action Plan’s decade of vaccines (2011–2020)^1^, disparities in vaccine coverage within and between countries persist,^3^ and the search for effective strategies to reach underserved populations has gained urgency.

Interventions to improve vaccination outcomes are commonly grouped into those targeting health services delivery or supply (e.g. improving human resources training, logistics, cold chain maintenance and vaccine storage, effective financing, monitoring and evaluation and supportive supervision) and those that stimulate demand for vaccines (e.g. monetary or food incentives, knowledge transfer or communication campaigns). Certain demand-side interventions have been associated with improved vaccine coverage of children in low- and middle-income countries in previous systematic reviews.^4^^–^^9^ However, limitations in study quality and design precluded quantitative synthesis in these reviews. The most recent review considered articles published up to 2009.^9^ In the interim, several new studies of potentially higher quality have been published.

We carried out a systematic review and meta-analysis to evaluate whether demand-side interventions increase uptake of routine childhood vaccination in low- and middle-income countries. Our objectives were to ascertain the effect of demand-side interventions on vaccine coverage and to identify which strategies are effective. We focused on demand-side interventions, since these strategies can more easily reach underserved populations and reduce inequities in immunization coverage.

## Methods

### Protocol and registration

The review protocol was registered in the PROSPERO database (CRD42013005783; available at: http://www.crd.york.ac.uk/PROSPERO/display_record.asp?ID=CRD42013005783). The full report, available from the authors, includes both a narrative synthesis and a meta-analysis as outlined in the protocol.

### Eligibility criteria

We defined six criteria for inclusion of studies in the review and meta-analysis: (i) based on guidelines developed by the Cochrane Effective Practice and Organisation of Care (EPOC) group, randomized controlled trial, non-randomized controlled trial, controlled before-after or interrupted time series study designs were eligible;^10^ (ii) the study location was a low- and middle-income country as defined by the World Bank;^11^ (iii) the study was published in a peer-reviewed scientific journal, because in previous reviews that considered the grey literature, the quality of these studies was found to be low;^4^^–^^7^ (iv) the participants were generally parents and caregivers of children younger than two years, living in communities where interventions to increase demand for routine childhood vaccination had been carried out; (v) eligible interventions were defined broadly as any intervention that might increase demand for routine childhood vaccination, such as incentives of money or food, knowledge transfer initiatives, or communication campaigns (studies that included strategies targeting vaccine supply in addition to demand were eligible); and (vi) the study outcomes included quantitative estimates of routine childhood vaccination coverage.

### Information sources

We searched the MEDLINE (via Pubmed), EMBASE, Cochrane library, POPLINE, ECONLIT, CINAHL, LILACS (Latin America and the Caribbean Center on Health Sciences Information), BDSP (French public health database), Web of Science and Scopus databases using appropriate terms and descriptors. Searches were limited to articles concerning human data that were written in English, French, German, Hindi, Portuguese or Spanish. The search began on 1 September 2008 and was last updated on 25 March 2014. All published studies included in previous systematic reviews^4^^–^^9^ were also considered. We consulted experts and reviewed article reference lists for additional articles.

### Search and selection

One of the authors and an academic librarian defined the MEDLINE search strategy (available from corresponding author). Search terms were combinations of “interventions”, “programs”, “approaches”, “subsidies”, “knowledge translation”, “vouchers”, “vaccination”, “immunization”, “vaccines”, “child”, “infant”, “newborn”, “kid”, and “low- and middle-income countries”. The author translated the strategy and terms for the databases in other languages. Identified records were uploaded into EndNote (Thomson Reuters, Philadelphia, United States of America) and duplicates eliminated. Two authors screened titles and abstracts independently and eliminated studies that failed to meet eligibility criteria. Full texts of remaining studies were retrieved. Two authors independently screened full text articles against study inclusion criteria and compared results; another author validated all decisions.

### Data extraction

From each study, two authors independently extracted data on study design, aims, location, population, intervention, follow-up period and outcomes, using a pre-defined template (available from corresponding author). We adopted the original study definitions of comparator or control groups. We pilot tested the template on a subset of studies. In addition to vaccine outcomes, information on equity and economic outcomes were extracted. Together, three authors crosschecked and verified these data. Study authors were contacted for clarification if data were missing or unclear.

### Risk of bias

We used Cochrane collaboration criteria to assess risk of bias.^12^ As these criteria were developed to assess randomized controlled trials, we supplemented them to accommodate additional study designs. To address issues specific to cluster randomized controlled trials, we systematically considered recruitment bias, unit of analysis bias and sample size as part of the “other” category.^12^ To accommodate non-randomized designs, we used the Cochrane EPOC group’s additional criteria and scoring for non-randomized controlled trials, controlled before-and-after and interrupted time-series studies.^10^ For each included study, two authors independently assessed risk of bias and compared results, and another author validated all decisions.

### Statistical analysis

The principal measure was the relative risk (RR) of vaccination among children in intervention versus control groups. We performed a meta-analysis by estimating random effect models with inverse variance weighting. This method gives greater weight to studies with more precise estimates. Study-specific estimates of variance were obtained by deriving standard errors from the confidence intervals reported by the studies. For cluster randomized controlled trials, we used the standard errors with adjustment for clustering, which allowed us to use both clustered and non-clustered randomized controls trials in the same meta-analyses. In addition to conducting meta-analysis on all included studies, we performed meta-analysis on five pre-specified subgroups: (i) studies including the third dose of diphtheria-tetanus-pertussis (DTP3); (ii) studies using knowledge transfer interventions; (iii) studies using incentives, (iv) studies whose risk of bias was assessed to be moderate-to-high; and (v) studies whose risk of bias was assessed to be low. We also estimated three meta-regression models adjusting separately for: (i) baseline vaccination coverage (the proportion of children aged 12–23 months receiving DTP3 in the study area or country); (ii) intervention type; and (iii) study quality. Heterogeneity was assessed using Cochrane’s *Q*-test and *I^2^* statistics.^12^ Potential publication bias was assessed using funnel plots. Analyses were performed using the metafor package^13^ for meta-analyses in R (R Foundation for Statistical Computing, Vienna, Austria) and the heterogi package^14^ for Stata (version 13.1, StataCorp LP, College Station, USA).

## Results

### Study selection

**Fig. 1:**
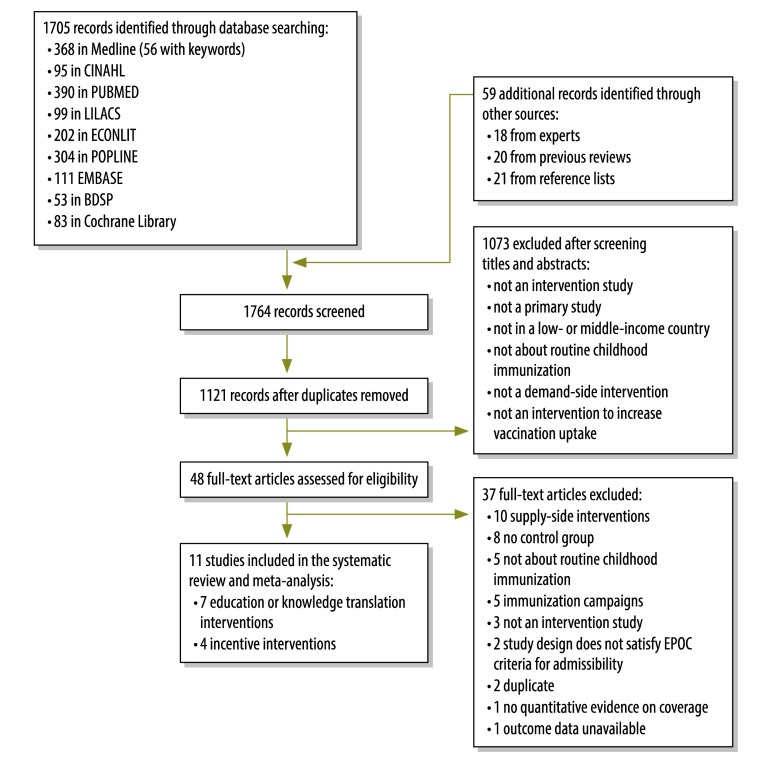
**Interventions to increase the demand for childhood vaccination: selection of studies**

Search of the databases yielded 1705 citations. We also identified 59 records through previous systematic reviews, article bibliographies and subject-matter experts. After removal of 643 duplicate records, there were 1121 records for title and abstract screening. Of these, 1073 did not meet eligibility criteria and were excluded. The full text of the remaining 48 articles was retrieved for detailed review (available from corresponding author). It lists the 37 articles excluded after full-text evaluation and the principal reasons for their exclusion. A total of 11 studies^15^^–^^25^ comprising four randomized controlled trials, six cluster randomized controlled trials and one controlled before-and-after study were included in the review (Fig. 1 and Table 1).

EPOC: Effective Practice and Organisation of Care.

CINAHL: Cumulative Index to Nursing and Allied Health Literature

PubMed: Public MEDLINE

LILACS: Latin-American and Caribbean Center on Health Sciences Information

ECONLIT: The American Economic Association’s electronic bibliography

POPLINE: Free resource from the John Hopkins Bloomberg School of Public Health

EMBASE: Elsevier’s biomedical database

BDSP : Banque de Données en Santé Publique

**Table 1:** Studies included in systematic review and meta-analysis of strategies to increase the demand for childhood vaccination in low- and middle-income countries

Study and year	Site	Study design	Participants	Intervention	Control	Vaccination Outcomes
Robertson et al., 2013^24^	Zimbabwe, rural areas	Cluster randomized controlled trial	30 areas were matched on socioeconomic characteristics and randomized to three groups (10 UCT: 10 CCT: 10 control). Households were eligible to participate if they lived in a study area and met need-based criteria. Vaccination outcomes consider children aged 0–4 years in participating households (934 in the intervention arms (517 UCT; 417 CCT) and 360 controls).	Unconditional cash transfer: every household collected US$ 18 plus US$ 4 per child (up to a maximum of three children) from designated pay points every 2 months. Conditional cash transfer: households received the same amount, but were monitored for compliance with several conditions. Among others, children less than 5 years had to be vaccinated on time.	No incentives	Proportion of children less than 5 years with up-to-date vaccinations (measles; BCG; polio; and diphtheria–tetanus–pertussis)
Briere et al., 2012^25^	Kenya, largely rural	Controlled before-and-after study	Comparison between two adjacent districts. Children aged 2–13 months were eligible; 1607 children in the intervention arm and 723 children in the control arm.	During routine immunization visits, caregivers with a child aged < 12 months were offered free hygiene kits (sodium hypochlorite solution for household water treatment, soap, pictorial educational materials) and education about water treatment and hand hygiene.	Routine immunization only	Children 2–13 months with up-to-date immunization coverage (3 doses of pentavalent vaccine at 6, 10 and 14 weeks and 1 dose of measles vaccine at 9 months)
Owais et al., 2011^15^	Pakistan, urban and peri-urban communities near Karachi	Randomized controlled trial	All mothers in five selected communities with a child ≤ 6 weeks old were eligible to participate; 179 children in the intervention arm and 178 in the control arm.	Short, home-based information sessions on importance of vaccines for low-literacy communities delivered by CHWs in 5 minutes. Pictorial cards conveyed three messages: vaccines save children’s lives; location of vaccination centres; and importance of retaining cards.	General health promotion messages (including information on vaccines) delivered by CHWs in 10–15 minutes	At four months after enrolment, children were defined as fully immunized if they completed all three doses of DTP3/Hepatitis B; otherwise non-immunized
Usman et al., 2011^17^	Pakistan, rural area near Karachi	Randomized controlled trial	All children visiting six selected EPI centres for DTP1 were eligible, if mothers had lived in the area for at least 6 months. Mother–child pairs were randomly allocated to 1 of 4 study groups; 1 128 participants in the intervention arms (Group 1: 378; 2: 376; 3: 374) and 378 in the control arm.	Group 1: A redesigned immunization card in a plastic jacket, with a hanging string. Group 2: 2–3 minutes conversation with mother during DPT1 visit to motivate and convey the potential adverse impact of incomplete immunization on the child’s health. Group 3: Received both interventions.	Mothers underwent routine EPI centre visits and received neither intervention	DTP3 completed (received both DTP2 and DTP3) versus DTP3 not completed
Banerjee et al., 2010^18^	India	Cluster randomized controlled trial	Within each of 134 randomly selected villages, 30 households with a child 0–5 years of age were randomly selected. Children were included if they belonged to a selected household and would be aged 1–3 years at the end of study (main sample) or were aged 0–6 months at baseline (baseline cohort). 761 children received interventions (A: 379; B: 382); 860 children served as controls.	Intervention A: A mobile team conducted monthly immunization camps in villages at fixed dates and times to improve services. In each village, a social worker performed outreach, linkage and educated mothers about immunization. Intervention B: Intervention A plus 1 kg of raw lentils per immunization and a set of metal plates for a child’s full immunization.	No intervention; had access to standard services	Children under 3 years who received ≥ 1 vaccine dose or were fully immunized;presence of BCG scar, number of immunizations, costs and cost-effectiveness
Andersson et al., 2009^19^	Pakistan, Lasbela district	Cluster randomized controlled trial	32 EAs were randomly selected.18 EAs (3166 children < 5 years) in intervention group. 14 EAs (2475 children < 5 years) in control group. Vaccination outcomes reflect a random sample of children aged 12–23 months in each cluster (intervention: 535 children; control: 422 children).	The intervention involved three structured discussions separately with male and female groups in each village. Discussions shared findings about local vaccine uptake; focused on the costs and benefits of childhood vaccination; and focused on local action plans. Participants spread the dialogue in their communities.	Access to standard immunization services; both groups received a district-wide health promotion programme on household hygiene	Proportions of children 12–23 months receiving DTP3 and measles vaccine; knowledge, attitudes and norms about vaccination; costs and cost-effectiveness
Usman et al., 2009^16^	Pakistan, urban area in Karachi	Randomized controlled trial	All children visiting five selected EPI centres for DTP1 were eligible to participate, if mothers had lived in the area for at least 6 months. Mother–child pairs were randomly allocated to 1 of 4 study groups; 1125 participants in the intervention arms (Group 1: 375; 2: 375; 3: 375) and 375 in the control arm.	Group 1: A redesigned immunization card in a plastic jacket, with a hanging string. Group 2: 2–3 minutes conversation with mother during DPT1 visit to motivate and convey the potential adverse impact of incomplete immunization on the child’s health. Group 3: Received both interventions.	Mothers underwent routine EPI centre visits and received neither intervention	DTP3 completed (received both DTP2 and DTP3) versus DTP3 not completed
Roy et al., 2008^23^	Bangladesh, rural	Randomized controlled trial	1275 poor women in 17 districts eligible for the rural maintenance programme were divided into three groups. Vaccination outcomes concerned 340 children 0–60 months (intervention: 126, control: 104, comparison group 110).	The standard programme provided income support, employment and skill training. The intervention group received basic nutrition and health education (including child immunization).	Comparison group received only standard programme; control group received neither programme nor education	Percentages of children 0–60 months receiving (by dose) DTP, measles, BCG, OPV that were partially vaccinated or fully vaccinated
Pandey et al., 2007^20^	India, Uttar Pradesh	Cluster randomized controlled trial	From 21 districts, 105 villages were randomly selected. 10 households per village (5 low caste, 5 middle-to-high caste) with at least one child going to public primary school were invited to join the baseline survey. Vaccine outcomes relate to 337 households (intervention 149; control 79) with a child less than 1 year	Campaigns to inform poor rural populations about entitled health and education services were conducted in two rounds in each village. Each round comprised two to three 1 hour meetings consisting of an audiotaped presentation, question period and leaflet distribution. Participants were re-interviewed after 12 months.	Access to standard services	Children less than 1 year old receiving ≥ 1 vaccine dose
Morris et al., 2004^21^	Honduras	Cluster randomized controlled trial	70 municipalities with the highest rates of malnutrition were selected and randomly assigned to one of four study groups in the ratio 2:1:2:2. Approximately 470 000 people received one or both interventions. Vaccination analyses for DTP1 compared 810 children in Group 1 to 878 controls.	Group 1 received vouchers worth £2.53 per month for each pregnant woman or child younger than 3 years, up to a maximum of two. Payments required compliance with child preventive health care. Group 2 improved health services via better planning, training and small repairs and purchases. Group 3 received both packages.	Access to standard services	Proportion of children 93 days to 3 years who received DTP1; proportion of children 1 year old who received measles vaccine
Brugha and Kevany, 1996^22^	Eastern Ghana	Cluster randomized controlled trial	A town with regular immunization services was subdivided into 30 matched pairs of clusters. One of each pair was randomly allocated to the intervention group. All 12–18 month old children living in intervention clusters joined the intervention arm (200 children); all 12–18 month old children residing in control clusters (219 children) joined the control arm.	Trained, non-health workers made home visits advising parents to bring their child to the next under-fives’ clinic. This advice was given to all respondents but targeted to parents of incompletely immunized children. Children who failed to complete the schedule following the referral were identified from a register and a nurse made up to three home visits over 6 months to each child.	Access to standard services	Proportions of children in each cluster who received polio 1, polio 3, measles, or were fully immunized (BCG, polio 3, DTP3 and measles)

BCG: bacille Calmette-Guérin; CCT: conditional cash transfer; CHW: community health worker; DTP1: diphtheria-tetanus-pertussis first dose; EA: enumeration area; EPI: expanded programme on immunization; OPV: oral polio vaccine; UCT: unconditional cash transfer; US$: United States dollars.

### Study characteristics

#### Study inclusion

The 11 studies were published in English between 1996 and 2013; eight were from lower-middle income countries^15^^–^^22^ and three were from low-income countries.^23^^–^^25^ Study locations included south Asia (*n* = 7),^15^^–^^20^^,^^23^ sub-Saharan Africa (*n* = 3),^22^^,^^24^^,^^25^ and central America (*n* = 1).^21^

#### Participants

Data were collected from 11 512 participants yielding outcomes for 11 512 children. As many interventions were directed to communities or populations rather than to individuals, the number of individuals reached by the interventions was considerably larger. Participants were mothers, caregivers, households of children who were within the target age-group for immunization (*n* = 9),^15^^–^^18^^,^^21^^–^^25^ or the general populations of target communities (*n* = 2).^19^^,^^20^

### Interventions

Of the 11 studies, seven described education or knowledge translation interventions,^15^^–^^17^^,^^19^^,^^20^^,^^22^^,^^23^ while four described interventions using incentives.^18^^,^^21^^,^^24^^,^^25^ Of the latter, two considered monetary incentives,^21^^,^^24^ and two non-monetary incentives.^18^^,^^25^ One study compared two types of monetary incentives;^24^ thus, the 11 studies yielded data on 12 interventions. Four studies considered both demand and supply interventions to improve vaccine coverage.^16^^–^^18^^,^^21^ For these studies, meta-analyses were based on the estimated demand-side effect.^16^^–^^18^^,^^21^

### Outcomes

Nine studies undertook coverage surveys to assess outcomes,^15^^,^^18^^–^^25^ while two used administrative data.^16^^,^^17^ For six studies, the main aim of the intervention was to increase immunization coverage,^15^^–^^19^^,^^22^ while for five studies, improving immunization coverage was a secondary aim and data on immunization outcomes were included.^20^^,^^21^^,^^23^^–^^25^

### Vaccination outcomes

Four studies described full immunization as defined by the country’s immunization schedule, all of which contain DTP3 as a subset.^18^^,^^22^^–^^24^ Six studies presented information on DTP3 vaccination.^15^^–^^17^^,^^22^^–^^24^ Three studies presented information on receipt of one or more vaccine doses^18^^,^^20^^,^^21^ and one considered age-appropriate vaccination.^25^ The timing of outcome measures was variable. While some studies addressed on-time delivery, a majority focused on the simpler assessment of presence or absence of vaccinations within a specified period. This period was based on the age of the child at the time outcomes were assessed and varied between studies: less than one year,^15^^–^^17^^,^^20^^,^^25^ less than two years,^19^^,^^22^ less than three years,^18^^,^^21^ or less than five years.^23^^,^^24^

One study recorded changes in immunization knowledge, attitudes and beliefs^19^ and two estimated intervention costs and cost–effectiveness,^18^^,^^19^ as additional vaccination-related outcomes. All studies considered equity in the choice of target populations by directing interventions to areas of greater need, but only two provided stratified analyses related to subgroups of interest.^17^^,^^20^

### Risk of bias

**Fig. 2:**
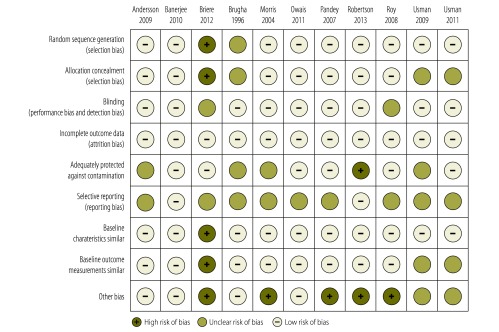
**Interventions to increase the demand for childhood vaccination: summary of the risk of study bias**

We assessed risk of bias for nine criteria. Our assessments ranged from low risk of bias on all criteria in one study^18^ to high risk of bias on five criteria in one study.^25^ For the purpose of subgroup analyses, we classified five studies with high risk of bias on one or more criteria as moderate-to-high risk of bias.^20^^,^^21^^,^^23^^–^^25^ The remaining six studies were categorised as low risk of bias.^15^^–^^19^^,^^22^ Fig. 2 presents a summary of our assessment of the risk of bias (a detailed assessment for each study and criterion, and figure summarizing risk of bias assessments by criterion is available from corresponding author) High risk of bias occurred most frequently for the category “other bias” (five studies), while unclear risk of bias was most frequent for the category “selective reporting” (nine studies). Risk of bias was related to intervention type: three of the four studies that used incentives had moderate-to-high risk of bias.^21^^,^^24^^,^^25^

### Receipt of vaccine

**Fig. 3:**
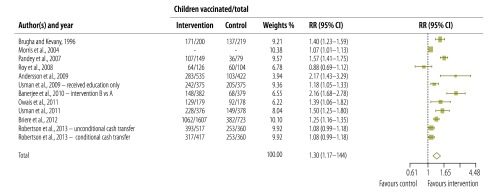
**Interventions to increase the demand for childhood vaccination: meta-analysis of 11 studies**

CI: confidence interval; RR: relative risk.

Note: Weights are from random-effects analysis.

RR estimates, 95% confidence intervals (CI) and weights for each individual study are shown in Fig. 3. For each study, the crude proportion of participants with and without vaccination is shown separately for intervention and control groups. For one study, Morris et al.,^21^ data necessary for meta-analysis were not available in the text or from the study author and were taken from a published article.^26^

In the pooled analysis considering all 12 interventions assessed in the 11 studies (Fig. 3), interventions were associated with significantly higher receipt of vaccines (RR: 1.30; 95% CI: 1.17–1.44), but there was considerable heterogeneity (*I^2^* = 88.96; 95% CI: 82.94– 92.16). Unweighted results were similar to weighted results (RR: 1.34; 95% CI: 1.21−1.50). To reduce diversity associated with outcomes measured, we considered only the nine studies reporting data for DTP3 or full vaccination (information available from corresponding authors). These results were similar to those for all studies (RR: 1.32; 95% CI: 1.15–1.51; *I^2^* = 86.31; 95% CI: 75.31–91.11).

### Bias across studies

To explore potential sources of heterogeneity, we plotted the observed outcome against the standard error for all meta-analyses and meta-regressions. Visual inspection of funnel plots revealed no evidence of asymmetry (available from corresponding author); however, due to the small number of studies the test has low power and we cannot exclude the possibility of publication bias.

### Subgroup analyses

The overall effect of educational or knowledge translation interventions was greater than for incentives RR: 1.40 (95% CI: 1.20–1.63) versus RR: 1.28 (95% CI: 1.12–1.45), respectively (available from corresponding author). The pooled analysis considering studies with a low risk of bias yielded a higher estimate of overall effect than that for studies with moderate-to-high risk of bias, RR: 1.53 (95% CI: 1.28−1.82) and RR: 1.15 (95% CI: 1.02–1.30), respectively (available from corresponding author). Heterogeneity was lower for educational or knowledge translation interventions: *I^2^* = 80.48, (95% CI: 53.90*–*88.88) than for incentives *I^2^* = 89.40, (95% CI: 76.83–93.73). Heterogeneity was also lower for studies with low risk of bias: *I^2^* = 79.16, (95% CI: 42.92−88.82) versus studies with moderate-to-high risk of bias: *I^2^* = 90.26, (95% CI: 81.40–93.86). Meta-regression revealed no important differences in results by baseline coverage level. Results for meta-regressions by intervention type and study quality were consistent with the corresponding meta-analyses.

## Discussion

This meta-analysis demonstrates that demand-side interventions lead to an increase in child vaccination coverage in diverse low- and middle-income settings and among communities with lagging health and social indicators. Immunization programmes have often focused on strengthening vaccine supply. Our results show that policymakers who seek to increase access to vaccines through the routine immunization system should also consider demand-side strategies.

Educational or knowledge translation interventions were more effective on average than interventions based on incentives; however, both strategies were effective. The highest estimates of effect and lowest heterogeneity were found among studies with low risk of bias. Risk of bias was related to intervention type, with three of the four incentives studies having moderate-to-high risk of bias.

Our analysis has six important limitations. First, vaccination coverage is shaped by interrelated supply and demand factors. Many studies, particularly those tackling vaccine and non-vaccine outcomes,^20^^,^^21^^,^^23^^–^^25^ did not give due consideration to the role of the health system in delivering vaccines during the study design phase leading to increased risk of bias. Three supply-side challenges confronted the studies reviewed: (i) increases in demand cannot be effective if supply-side constraints limit provision of vaccines;^21^^,^^25^ (ii) if coverage rates are already very high, there is limited scope to demonstrate improvement;^24^^,^^25^ and (iii) vaccination rates can be affected by changes in service delivery occurring independently of the study. Among the studies reviewed, only one study did not show a gain associated with the intervention: in this study, all experimental groups experienced substantial but similar gains in vaccination coverage.^23^ Another study noted a likely background trend with a differential impact on experimental groups.^25^ Unfortunately, neither of these studies collected data on health system trends that might affect immunization delivery. Studies addressing vaccine and non-vaccine outcomes, including three of the four incentive based studies^21^^,^^24^^,^^25^ may have faced challenges related to broad study scope or a lack of vaccine-specific expertise in study planning. In addition, two studies of financial incentives^21^^,^^24^ were large-scale interventions and faced substantial implementation challenges.

Second, the systematic review and meta-analysis included only 11 studies. This limited our ability to explore potential sources of heterogeneity quantitatively and to exclude possible biases related to publication and study size.

Third, studies measured different vaccines over different time periods. Receipt of DTP3 is the measure preferred by international agencies to assess immunization coverage and was included in nine of the 11 papers reviewed.^3^ All analyses consistently showed that demand-side interventions are beneficial in improving coverage. However, due to diversity in outcomes and the small number of studies, we cannot evaluate the effect of interventions for specific vaccines.

Fourth, health gains depend not only on increased vaccine coverage but also on appropriate timing of vaccination. With one exception,^25^ studies offered little information on when doses were delivered.

Fifth, despite contacting the authors, we were not able to retrieve data for all studies and had to use approximate values for one study^21^ based on another publication.^26^

Sixth, our analysis excluded interventions that focused exclusively on improving vaccine supply. However, from a policy point of view, how demand-side interventions interact with supply side constraints is also important. With one exception,^18^ immunization system performance was not explicitly assessed by these studies.

Our results indicate that future research on demand-side interventions to increase vaccine coverage should (i) standardize measurement of outcomes; (ii) include vaccination experts during the study design phase; and (iii) collect data on health system characteristics that may affect vaccine delivery.

## Conclusion

Demand-side interventions are effective in improving the uptake of childhood vaccines delivered through routine immunization services in low- and middle-income countries. Our results are more definitive than those of previous systematic reviews which employed narrative synthesis techniques.^4^^–^^9^

Demand-side strategies to improve vaccination coverage are important because they are inherently equity-oriented and address specific barriers to coverage related to financial constraints, opportunity costs, knowledge and prioritization. Future research should seek to refine our understanding of which approaches are most effective in specific contexts. Studies investigating the value of knowledge translation and incentives-based interventions offered in combination are also required. Studies that simultaneously consider supply- and demand-side interventions – and enable us to evaluate their relative effectiveness – are of particular interest. Finally, studies should consider whether interventions can be delivered effectively at scale and in the long term.

## References

[1] Decade of Vaccines Collaboration 2012. Global Vaccine Action Plan 2011-2020. Geneva: World Health Organization; 2013. Available from: http://www.who.int/iris/bitstream/10665/78141/1/9789241504980_eng.pdf?ua=1 [cited 2015 March 3].

[2] Narrowing the gaps to meet the goals. New York: United Nations Children’s Fund; 2010. Available from: http://www.unicef.org/publications/files/Narrowing_the_Gaps_to_Meet_the_Goals_090310_2a.pdf [cited 2015 Mar 23].

[3] Global immunization data. Geneva: World Health Organization; 2014. Available from: http://www.who.int/immunization/monitoring_surveillance/global_immunization_data.pdf [cited 2015 Mar 23].

[4] Batt K, Fox-Rushby JA, Castillo-Riquelme M. The costs, effects and cost-effectiveness of strategies to increase coverage of routine immunizations in low- and middle-income countries: systematic review of the grey literature. Bull World Health Organ. 2004 9;82(9):689–96. 15628207 PMC2622984

[5] Pegurri E, Fox-Rushby JA, Damian W. The effects and costs of expanding the coverage of immunisation services in low- and middle-income countries: a systematic literature review. Vaccine. 2005 2 18;23(13):1624–35. 10.1016/j.vaccine.2004.02.029 15694515

[6] Haines A, Sanders D, Lehmann U, Rowe AK, Lawn JE, Jan S, et al. Achieving child survival goals: potential contribution of community health workers. Lancet. 2007 6 23;369(9579):2121–31. 10.1016/S0140-6736(07)60325-0 17586307

[7] Ryman TK, Dietz V, Cairns KL. Too little but not too late: results of a literature review to improve routine immunization programs in low- and middle-income countries. BMC Health Serv Res. 2008;8(1):134. 10.1186/1472-6963-8-134 18570677 PMC2474611

[8] Shea B, Andersson N, Henry D. Increasing the demand for childhood vaccination in low- and middle-income countries: a systematic review. BMC Int Health Hum Rights. 2009;9 Suppl 1:S5. 10.1186/1472-698X-9-S1-S5 19828063 PMC3226237

[9] Oyo-Ita A, Nwachukwu CE, Oringanje C, Meremikwu MM. Interventions for improving coverage of child immunization in low- and middle-income countries. Cochrane Database Syst Rev. 2011; (7):CD008145. 21735423 10.1002/14651858.CD008145.pub2

[10] Effective Practice and Organisation of Care (EPOC). What study designs should be included in an EPOC review and what should they be called? EPOC Resources for review authors. Oslo: Norwegian Knowledge Centre for the Health Services; 2015. Available from: http://epoc.cochrane.org/epoc-specific-resources-review-authors [cited 2015 Mar 23].

[11] Country and lending groups. Data & statistics: country classification. Washington: The World Bank; 2013.

[12] Higgins JPT, Green S, editors. Cochrane handbook for systematic reviews of interventions. Oxford: The Cochrane Collaboration; 2011. Available from: http://handbook.cochrane.org/ [cited 2015 April 10].

[13] Viechtbauer W. Conducting meta-analyses in R with the metafor package. J Stat Softw. 2010;36:1–48.

[14] Higgins JP, Thompson SG. Quantifying heterogeneity in a meta-analysis. Stat Med. 2002 6 15;21(11):1539–58. 10.1002/sim.1186 12111919

[15] Owais A, Hanif B, Siddiqui AR, Agha A, Zaidi AK. Does improving maternal knowledge of vaccines impact infant immunization rates? A community-based randomized-controlled trial in Karachi, Pakistan. BMC Public Health. 2011;11(1):239. 10.1186/1471-2458-11-239 21496343 PMC3094245

[16] Usman HR, Akhtar S, Habib F, Jehan I. Redesigned immunization card and center-based education to reduce childhood immunization dropouts in urban Pakistan: a randomized controlled trial. Vaccine. 2009 1 14;27(3):467–72. 10.1016/j.vaccine.2008.10.048 18996423

[17] Usman HR, Rahbar MH, Kristensen S, Vermund SH, Kirby RS, Habib F, et al. Randomized controlled trial to improve childhood immunization adherence in rural Pakistan: redesigned immunization card and maternal education. Trop Med Int Health. 2011 3;16(3):334–42. 10.1111/j.1365-3156.2010.02698.x 21159080 PMC3763701

[18] Banerjee AV, Duflo E, Glennerster R, Kothari D. Improving immunisation coverage in rural India: clustered randomised controlled evaluation of immunisation campaigns with and without incentives. BMJ. 2010 5 17;340(1):c2220. 10.1136/bmj.c2220 20478960 PMC2871989

[19] Andersson N, Cockcroft A, Ansari NM, Omer K, Baloch M, Ho Foster A, et al. Evidence-based discussion increases childhood vaccination uptake: a randomised cluster controlled trial of knowledge translation in Pakistan. BMC Int Health Hum Rights. 2009;9 Suppl 1:S8. 10.1186/1472-698X-9-S1-S8 19828066 PMC3226240

[20] Pandey P, Sehgal AR, Riboud M, Levine D, Goyal M. Informing resource-poor populations and the delivery of entitled health and social services in rural India: a cluster randomized controlled trial. JAMA. 2007 10 24;298(16):1867–75. 10.1001/jama.298.16.1867 17954538

[21] Morris SS, Flores R, Olinto P, Medina JM. Monetary incentives in primary health care and effects on use and coverage of preventive health care interventions in rural Honduras: cluster randomised trial. Lancet. 2004 12 4-10;364(9450):2030–7. 10.1016/S0140-6736(04)17515-6 15582060

[22] Brugha RF, Kevany JP. Maximizing immunization coverage through home visits: a controlled trial in an urban area of Ghana. Bull World Health Organ. 1996;74(5):517–24. 9002332 PMC2486871

[23] Roy SK, Bilkes F, Islam K, Ara G, Tanner P, Wosk I, et al. Impact of pilot project of Rural Maintenance Programme (RMP) on destitute women: CARE, Bangladesh. Food Nutr Bull. 2008 3;29(1):67–75. 18510207 10.1177/156482650802900108

[24] Robertson L, Mushati P, Eaton JW, Dumba L, Mavise G, Makoni J, et al. Effects of unconditional and conditional cash transfers on child health and development in Zimbabwe: a cluster-randomised trial. Lancet. 2013 4 13;381(9874):1283–92. 10.1016/S0140-6736(12)62168-0 23453283 PMC3627205

[25] Briere EC, Ryman TK, Cartwright E, Russo ET, Wannemuehler KA, Nygren BL, et al. Impact of integration of hygiene kit distribution with routine immunizations on infant vaccine coverage and water treatment and handwashing practices of Kenyan mothers. J Infect Dis. 2012 3;205 Suppl 1:S56–64. 10.1093/infdis/jir779 22315387

[26] Bassani DG, Arora P, Wazny K, Gaffey MF, Lenters L, Bhutta ZA. Financial incentives and coverage of child health interventions: a systematic review and meta-analysis. BMC Public Health. 2013;13 Suppl 3:S30. 10.1186/1471-2458-13-S3-S30 24564520 PMC3847540

